# Anti-Epstein–Barr Virus Activities of Flavones and Flavonols with Effects on Virus-Related Cancers

**DOI:** 10.3390/molecules30051058

**Published:** 2025-02-26

**Authors:** Sherif T. S. Hassan

**Affiliations:** Department of Applied Ecology, Faculty of Environmental Sciences, Czech University of Life Sciences Prague, Kamýcká 129, 165 00 Prague, Czech Republic; sherif.hassan@seznam.cz

**Keywords:** antiviral properties, EBV-associated cancers, EBV life cycle, anticancer effects, Epstein–Barr virus, flavonoids, flavones, flavonols, host–EBV interaction, viral and cellular genes, viral and cellular proteins

## Abstract

The Epstein–Barr virus (EBV), a member of the human gamma-herpesviruses, is intricately linked to various human malignancies. Current treatment options for EBV infection involve the use of acyclovir and its derivatives, which exhibit limited efficacy and are associated with drug resistance issues. Therefore, there is a critical need for new medications with more effective therapeutic actions and less susceptibility to resistance. This review explores the therapeutic promise of flavones and flavonols, naturally occurring molecules, against EBV and its correlated cancers. It thoroughly delves into the molecular mechanisms underlying the therapeutic efficacy of these compounds and scrutinizes their complex interplay in EBV-linked processes and cancer transformation by targeting key genes and proteins pivotal to both the viral life cycle and tumor development. Additionally, the review covers current research, highlights key findings, and discusses promising avenues for future investigations in the pursuit of targeted therapies against EBV and its related tumors.

## 1. Introduction

The Epstein–Barr Virus (EBV), a member of the herpesvirus family, is a pervasive human pathogen that infects the majority of the world’s population [1,2]. It is classified as a tumor gamma-herpesvirus (human herpesvirus 4) and is transmitted through saliva, causing infectious mononucleosis, colloquially known as the “kissing disease” [3,4]. EBV establishes a lifelong latent infection in B cells (B lymphocytes), with the potential for reactivation during periods of stress or immunosuppression [5,6]. While EBV infection is often asymptomatic, its implications become significant in the context of certain malignancies. This virus has been linked to the development of various cancers, including Burkitt’s lymphoma, Hodgkin’s lymphoma, nasopharyngeal carcinoma (NPC), extranodal natural killer (NK)/T-cell lymphoma (ENKTCL), leiomyosarcomas, and gastric carcinoma [7,8,9,10]. Furthermore, EBV profoundly affects the immune system and is a common agent of fatal lymphoproliferative disorders in immunosuppressive conditions. Moreover, it is recognized as a major contributor to multiple sclerosis [11,12,13].

Epidemiological evidence robustly establishes a link between chronic EBV infection and an elevated risk of various cancers, as mentioned above. Central to this association are the latent genes expressed by EBV during persistent infection, such as Epstein–Barr nuclear antigen (EBNA) proteins, latent membrane proteins (LMPs), and noncoding RNAs such as EBV-encoded small RNAs (EBERs) [14,15,16]. These viral components intricately interact with cellular signaling pathways, modulating cell growth, apoptosis, immune evasion, and genomic stability, thereby fueling the initiation and progression of EBV-associated malignancies. Mechanistically, EBNA proteins facilitate cell cycle progression and inhibit apoptosis, while LMPs activate key signaling cascades such as nuclear factor-κB (NF-κB) and phosphoinositide 3-kinase/protein kinase B (PI3K/Akt), fostering cell proliferation and resistance to cell death [17,18,19,20]. Additionally, EBV-encoded microRNAs (miRNAs) and EBERs exert regulatory control over gene expression and immune responses, further driving oncogenic processes [21,22]. Insight into the role of EBV latent genes in tumorigenesis not only advances our understanding of EBV-associated oncogenesis but also holds promise for the development of targeted therapeutic strategies aimed at disrupting viral gene expression or their downstream cellular pathways [23,24].

The primary treatment paradigm for EBV centers on symptomatic management, given the absence of a targeted antiviral cure [25,26]. Prescribed medications such as acyclovir, valacyclovir, and famciclovir, though exhibiting limited efficacy against EBV, are employed. While these medications do not eradicate the virus, they play a crucial role in alleviating infection symptoms and potentially abbreviating the duration of the viral outbreak. However, drug resistance issues have been raised with their overuse [27,28,29]. In some cases, corticosteroids may also be used to reduce inflammation and swelling associated with severe symptoms of infectious mononucleosis, although their use is not recommended for children presenting common infection symptoms [30,31]. However, due to these hurdles and the limitations of current treatments, there is a pressing need to explore and identify effective cures for EBV and its linked cancers. Flavonoids emerge as promising candidates in this pursuit, presenting a potential avenue for more targeted and efficient therapeutic interventions [32,33].

The aim of this review is to explore and analyze the potential of flavones and flavonols as precision-targeted therapeutic agents against EBV and its associated malignancies. The focus is on understanding how these compounds intervene in the EBV life cycle by targeting key genes and proteins crucial for both viral replication and tumor development. The review seeks to unravel the intricate mechanisms through which flavones and flavonols contribute to inhibiting EBV-associated tumors. Additionally, it aims to provide a comprehensive overview of experimental approaches, including in vitro, in vivo, and in silico studies, to strengthen the evidence supporting the feasibility and effectiveness of this precision-targeting strategy.

The literature search strategy commenced with the utilization of major databases, including Scopus, Web of Science Core Collection, PubMed, ScienceDirect, Google Scholar, SciFinder, and ClinicalTrials.gov. I employed specific keywords related to flavones and flavonols with anti-EBV activities, focusing on known mechanisms of action, specifically targeting genes and proteins essential for the viral life cycle and tumor progression. The collected data were extracted from studies published between 2012 and 2023. To ensure rigorous comparison, analysis, and evaluation, some studies published before 2012 were included.

## 2. A Brief Overview of the EBV Life Cycle

EBV is a type of double-stranded DNA virus characterized by approximately 100 protein-coding genes, as well as numerous noncoding RNAs and microRNAs (miRNAs) [34,35,36]. It undergoes a multifaceted life cycle predominantly within B cells of the human immune system. The process initiates when the virus attaches to host cells and enters them through viral proteins such as gp350/gp220 and gp42. Subsequently, fusion occurs using glycoproteins (gB, gH, and gL), facilitating the release of the viral capsid into the host cell [37,38,39,40]. This marks the commencement of the lytic phase, characterized by the transcription of viral DNA, synthesis of capsid proteins, and the assembly of new virions within the cell nucleus. The lytic phase concludes with cell rupture, enabling the spread of the virus to neighboring cells. In addition to the lytic phase, EBV can establish latent infections, wherein the viral genome persists in B cells, remaining dormant until sporadic reactivation prompts the return to the lytic phase [41,42,43,44]. Notably, EBV employs various immune evasion strategies, including targeting the major histocompatibility complex class I (MHC-I) and MHC-II expressions [45,46,47,48]. Understanding this complex life cycle is critical for addressing associated diseases, including infectious mononucleosis and certain cancers such as Burkitt’s lymphoma, Hodgkin’s lymphoma, nasopharyngeal carcinoma, gastric carcinoma, ENKTCL, and leiomyosarcomas [49,50,51].

## 3. Flavones and Flavonols: Chemistry, Antiviral, and Anticancer Properties

Flavones and flavonols are subclasses of flavonoids, a diverse group of polyphenolic compounds present in various fruits, vegetables, and plant-based foods [52,53]. These compounds serve multiple roles in plants, including pigmentation, UV protection, and defense against pathogens and abiotic stresses [54,55,56]. Both flavones and flavonols share a common structure, consisting of two aromatic rings (A and B rings) connected by a three-carbon bridge (C ring). Additionally, they both feature hydroxyl groups at various positions on the A and B rings. Furthermore, flavones and flavonols can exist in the form of glycosides [57,58,59]. Biologically, they have been observed to exhibit a diverse range of activities [60,61,62]. They are known to suppress the activity of various DNA and RNA viruses associated with cancers. Their potential antiviral and anticancer properties stem from their ability to interact with key molecular targets in both viral life cycles and tumor development pathways [32,63,64,65,66,67].

## 4. Targeting the EBV Life Cycle by Flavones and Flavonols

**Figure 1:**
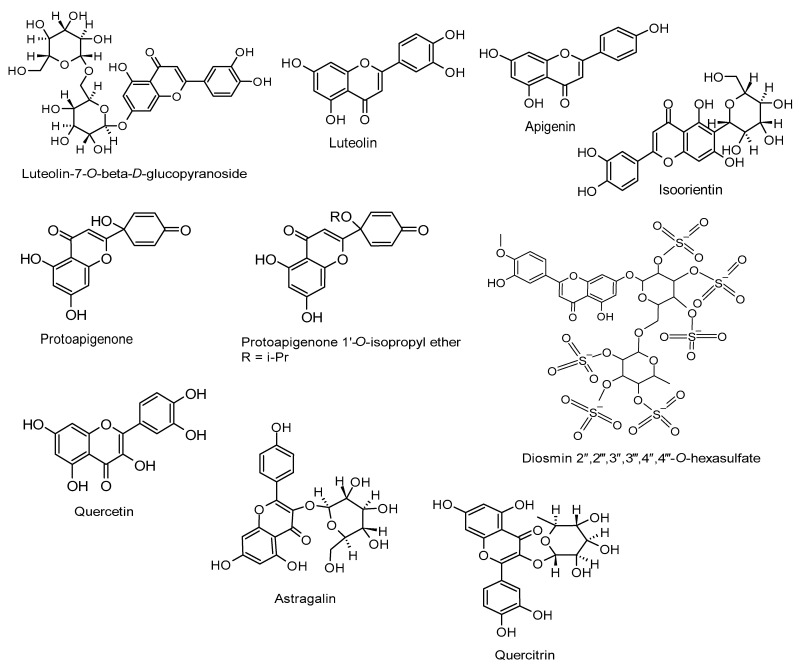
Chemical structures of flavones and flavonols with anti-EBV properties.

Understanding the importance of targeting critical genes and proteins crucial to the EBV life cycle presents a promising approach for developing effective antiviral drugs [32,68,69]. Table 1 offers a comprehensive investigation of flavones and flavonols as potential inhibitors of the EBV life cycle by disrupting various stages, detailing their mechanisms of action. Figure 1 further elucidates the chemical structures of these inhibitory molecules. As shown in the table, these compounds interfere with key processes such as viral entry, lytic replication, DNA load, virion production, and latency through their interactions with essential gene and protein expressions. These targets include the replication and transcription activator (Rta), immediate-early gene (Zta), EBV early antigen (EBV-EA), latent membrane protein 1 (LMP1), transcription factor specificity protein 1 (SP1), viral capsid antigen (VCA), early antigen D (EA-D), and Epstein–Barr nuclear antigen 1 (EBNA1).

## 5. Targeting EBV-Associated Tumors by Flavones and Flavonols

Throughout different phases of the EBV life cycle, a diverse array of genes, proteins, and antigens are expressed, responsible for lytic activation, latency, reactivation, and tumor development. These expressed entities play crucial roles in cellular processes such as growth, transformation, and metabolic reprogramming, thereby enhancing the oncogenic capabilities of EBV. Hence, approaches involving interventions targeted at disrupting the lytic cycle and regulating the expression of lytic and latent genes, proteins, and antigens are essential to manage EBV-associated tumors. Intervention strategies, such as the use of flavones and flavonols, which have shown promise in targeting specific stages of the EBV life cycle, further underscore their ability to combat EBV-related malignancies through interaction with EBV gene, protein, and antigen products [16,32,79,80,81].

Luteolin, a bioactive flavone present in various medicinal plants, fruits, and vegetables, emerges as a promising therapeutic agent against EBV-associated NPC, acting on crucial molecular pathways in cancer development and viral lytic replication. It demonstrates the ability to suppress EBV reactivation, key gene and protein expressions, and genomic instability in NPC cells. Moreover, luteolin exhibits inhibitory effects on cell proliferation, migration, invasion, and overall tumor growth in mouse models [82]. In its targeted approach to EBV-LMP1, a significant driver of NPC cell proliferation and development, luteolin hampers lipogenesis and cell growth by effectively suppressing the expressions of LMP1, sterol regulatory element-binding protein 1 (SREBP1), and fatty acid synthase (FASN). This multifaceted impact extends beyond inhibiting NPC growth to inducing apoptosis in mice [83]. Additionally, luteolin showcases its prowess in impeding EBV infection through lytic replication inhibition. This involves precise targeting of Rta and Zta expression and the downregulation of SP1 activity, leading to the suppression of NPC growth [71].

Wogonin, a flavone derived from *Scutellaria baicalensis*, exhibits anti-EBV lymphoma properties in both in vitro and animal models. It induces cell apoptosis by suppressing the NF-κB pathway through modulation of the LMP1/miR-155/NF-κB/PU.1 axis. Moreover, in mouse xenograft models with EBV-infected lymphoma, wogonin hinders tumor growth by inhibiting ki67 and p65 expression [84].

Baicalein, a flavone extracted from *Scutellaria baicalensis*, effectively reduces the expressions of Sp1, EBNA1, and EBNA1 Q-promoter in EBV-positive NPC cells. Notably, in a mouse xenograft model of EBV-positive NPC, baicalein significantly inhibits tumor progression [85]. Furthermore, when targeting EBV-positive B-cell tumors, this substance activates the apoptosis signal-regulating kinase 1/c-Jun N-terminal kinase (ASK1/JNK) pathway and regulates mitochondria-dependent apoptosis. This involves modulating the expression of transcriptionally active p63 (TAp63) and downregulating NF-κB, CD74, and CD44 proteins [86].

Quercetin, a bioactive flavonol found in numerous fruits, vegetables, and medicinal herbs, displays multiple health benefits. Its efficacy extends to combating diverse diseases, including EBV-associated malignancies, as demonstrated through various mechanisms validated by laboratory and animal experiments. In a mice xenograft model of EBV-gastric carcinoma, quercetin demonstrated a potent anticancer effect. The molecular mechanism underlying this action involved the modulation of viral EBNA1 and LMP2 expression. Furthermore, quercetin induced p53-dependent apoptosis in EBV-gastric carcinoma, as indicated by increased expression of cleaved forms of caspase-3, -9, and Parp [87]. In another animal experiment, the synergy with *Ganoderma lucidum* extract further heightened quercetin’s antitumor activity. This combined approach showcased enhanced effectiveness in treating EBV-gastric carcinoma [88]. In another research study, quercetin’s efficacy in inducing cytotoxic effects, apoptosis, and cell cycle arrest on EBV-gastric carcinoma cells was evident. Notably, it hindered EBV infection by targeting viral entry and latency while concurrently inhibiting EBNA1 expression [77]. In addition to gastric carcinoma, quercetin demonstrated inhibitory potency against EBV-positive Burkitt’s lymphoma cells by downregulating cellular myelocytomatosis oncogene (c-Myc) expression, leading to apoptosis [89]. Additionally, its preventive role in EBV-positive NPC cells was evident in vitro via inhibiting cell proliferation and reducing FASN expression [90]. In an in vitro exploration, Granato et al. [91] delved into the robust anti-tumor potential of quercetin against EBV-associated lymphomas. They focused on targeting interleukin-6 (IL-6) and other oncogenic pathways essential for fostering viral carcinogenesis.

The investigation into the potential of icaritin, a prenylated flavonol derived from the *Epimedium* genus, against EBV-positive ENKTCL, a highly aggressive hematological tumor, revealed that it exerts anti-proliferative and pro-apoptotic effects on ENKTCL cells. It influences crucial apoptotic proteins and disrupts cell cycle progression. The underlying mechanism involves the suppression of signal transducer and activator of transcription 3 (STAT3) and Akt pathways through LMP1 downregulation. Notably, the combination of icaritin with the antiviral drug ganciclovir exhibited a potent induction of ENKTCL cell apoptosis. These findings highlight icaritin as a promising therapeutic candidate for EBV-associated ENKTCL, suggesting potential synergies when combined with antiviral drugs [92].

Fisetin, a flavonol present in diverse fruits, vegetables, and medicinal plants, demonstrates notable dietary and pharmacological attributes in addressing various health conditions. Research suggests its efficacy against the metastasis of EBV-associated NPC by suppressing the migration and invasion of LMP1-expressing cells. Fisetin achieves this action by curtailing molecular changes associated with epithelial–mesenchymal transition (EMT), boosting the expression of the epithelial marker E-cadherin, and reducing the levels of mesenchymal markers such as vimentin and twist proteins [93]. Furthermore, fisetin disrupts the NF-κB signal transduction pathway targets (p65, IκBα, and cyclinD1) activated by EBV-LMP1, as evidenced by in vitro studies on EBV-infected NPC cells [94].

Yun and colleagues [95] investigated dihydromyricetin, also known as ampelopsin, a dihydroflavonol compound from *Ampelopsis grossedentata*, against EBV-positive Burkitt’s lymphoma cells. This compound induced apoptosis and impeded cell proliferation by targeting various signaling pathways, including those associated with EBV-LMP1.

**Figure 2:**
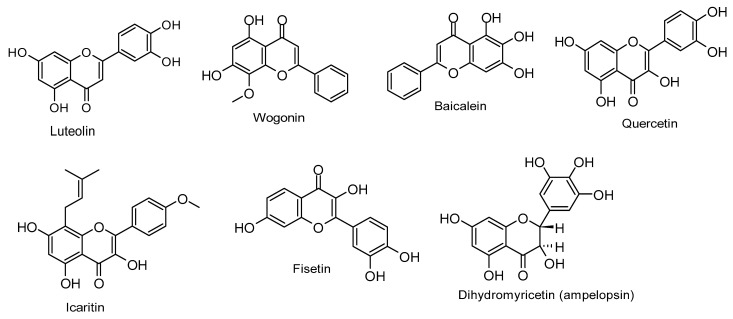
Chemical structures of flavones and flavonols with anti-EBV-associated cancers effects.

Table 2 illustrates the anti-EBV-related tumor activities of flavones and flavonols, outlining their mechanisms of action, while Figure 2 depicts their chemical structures.

## 6. Safety and Toxicity Considerations

The dual antiviral and anticancer properties of flavones and flavonols add a significant dimension to their potential health benefits [96,97]. Examining the safety and toxicity considerations of these natural compounds is crucial in harnessing their therapeutic potential. While these substances demonstrate promising health benefits, it is essential to acknowledge that, like any bioactive substance, they may have potential side effects [98,99,100]. Studies have generally reported low toxicity levels of flavones and flavonols, particularly when obtained from dietary sources. However, isolated and concentrated forms, such as supplements, may pose risks at higher doses [101,102]. Individual responses to these compounds can vary, and interactions with medications should be carefully evaluated. Additionally, exploring the impact of long-term exposure and bioavailability is vital for a comprehensive safety assessment [103,104,105]. Overall, a nuanced understanding of the dose–response relationship and individual variations is essential to strike a balance between reaping the benefits of flavones and flavonols and mitigating potential risks for optimal safety for therapeutic applications [106,107].

## 7. Conclusions, Challenges, and Future Directions

In conclusion, this review illuminates the promising of flavones and flavonols in precisely targeting EBV and its associated malignancies. By focusing on genes and proteins crucial to both the viral life cycle and cancer development, these compounds showcase a dual therapeutic effect. The ability to disrupt key processes in the EBV life cycle, coupled with the impact on cancer-related pathways, positions flavones and flavonols as promising candidates for the development of targeted therapies. The multifaceted nature of these natural compounds allows for a nuanced approach, addressing both the viral infection and the subsequent oncogenic transformation. Moreover, their demonstrated safety profiles and accessibility from natural sources further enhance their attractiveness as potential therapeutics.

However, several challenges persist in this field, particularly when addressing EBV and its associated tumors. The heterogeneity of EBV-associated cancers poses a substantial obstacle, demanding tailored strategies for different manifestations. Additionally, issues related to bioavailability, dosage optimization, and potential off-target effects of flavones and flavonols need careful consideration, specifically in the context of EBV and its linked cancers. Addressing these challenges is crucial for translating promising preclinical findings into effective clinical applications.

Looking ahead, future research should focus on refining the specificity and efficacy of flavones and flavonols against EBV and its related tumors. Investigating the potential synergistic effects of these compounds with existing therapies could enhance treatment outcomes. Moreover, exploring novel delivery mechanisms and formulations to improve bioavailability is a promising avenue for overcoming current limitations. Collaborative efforts between researchers and clinicians will be instrumental in conducting robust clinical trials to validate the therapeutic potential of flavones and flavonols, ultimately paving the way for the development of targeted and effective interventions against EBV and its linked malignancies.

## Figures and Tables

**Table 1:** Flavones and flavonols exert anti-EBV effects by disrupting critical genes and proteins involved in the viral life cycle.

Compound and Source	Classification	Study Type and Cells	Mechanism of Action (Inhibition/Downregulation)	Effective Concentration	Refs.
Luteolin-7-*O*-beta-*D*-glucopyranoside *Lindernia Crustacea*	Flavone	In vitro EBV-Burkitt’s lymphoma cells	Lytic replication Rta expression	20 µg/mL	[70]
Luteolin Diverse medicinal plants, fruits, and vegetables	Flavone	In vitro EBV-Burkitt’s lymphoma cells and EBV-NPC cells	Lytic replication Rta, Zta, and Sp1 expressions	10, 20, and 50 µM	[71]
Apigenin Fruits and vegetables	Flavone	In vitro EBV-epithelial cells	Lytic replication Rta and Zta expressions Virion production	50 µM	[72]
Isoorientin *Passiflora edulis*	Flavone	In vitro B16 mouse melanoma cells	Lytic cycle EBV-EA	IC_50_ = 393 mol ratio/32 pmol TPA	[73]
Diosmin 2″,2‴,3″,3‴,4″,4‴-*O*-hexasulfate Chemically modified form of diosmin	Flavone	In vitro and in silico EBV-Burkitt’s lymphoma cells	Lytic replication and LMP1 expression (in vitro) Zta (in silico)	20 µM (in vitro) −8.7 kcal/mol (in silico)	[74]
Protoapigenone *Thelypteris torresiana*	Protoflavone	In vitro EBV-Burkitt’s lymphoma cells	Lytic replication Rta, Zta, EA-D, and VCA expressions	IC_50_ = 0.127 µM 0.31 mM and 0.50 µM	[75,76]
Protoapigenone 1′-*O*-isopropyl ether Produced synthetically from Apigenin	Protoflavone	In vitro EBV-Burkitt’s lymphoma cells	Lytic replication Rta expression	IC_50_ = 0.467 µM 0.25 µM	[76]
Quercetin *Glycyrrhiza uralensis*	Flavonol	In vitro EBV-SNU719 cells	Entry and latent phases. EBNA1 expression	62 µM	[77]
Astragalin and quercitrin *Humulus lupulus*	Flavonol glycosides	In vitro EBV-Burkitt’s lymphoma cells	Lytic cycle EBV-EA expression	IC_50_ values of 543 and 532 mol ratio/32 pmol TPA, respectively	[78]

Abbreviations: EA-D, early antigen D; EBNA1, Epstein–Barr nuclear antigen 1; EBV, Epstein–Barr virus; EBV-EA, EBV early antigen; EBV-SNU719 cells, human gastric carcinoma cells infected with EBV; IC_50_, 50% inhibitory concentration; LMP1, latent membrane protein 1; NPC, nasopharyngeal carcinoma; Rta, replication and transcription activator; SP1, transcription factor specificity protein 1; TPA, 12-*O*-tetradecanoylphorbol-13-acetate; VCA, viral capsid antigen; Zta, an immediate-early gene.

**Table 2:** Flavones and flavonols exhibit anti-EBV-associated cancer properties by targeting essential genes and proteins crucial for tumor transformation and development.

Compound and Source	Classification	Study Type and Cancer Cells	Mechanism of Action (Inhibition/Downregulation)	Effective Concentration/Dose	Refs.
Luteolin Diverse medicinal plants, fruits, and vegetables	Flavone	In vitro and in vivo EBV-positive NPC cells	EBV reactivation Rta and Zta expressions Genomic instability, cell proliferation, migration, invasion, and spheroid formation	0–50 µM (in vitro). 40 mg/kg every 3 or 4 days for 4 weeks (in vivo)	[82]
		In vitro and in vivo EBV-positive NPC cells	Lipogenesis and proliferation of NPC cells LMP1, SREBP1, and FASN expressions	20 µM (in vitro) 20 mg/kg every 2–3 days for 3 weeks (in vivo)	[83]
		In vitro EBV-positive NPC cells	EBV lytic replication Tumor growth Rta, Zta, and SP1 expressions	10, 20, and 50 µM	[71]
Wogonin *Scutellaria baicalensis*	Flavone	In vitro and in vivo EBV-infected lymphoma cells	Tumor growth LMP1/miR-155/NF-κB/PU.1 pathway ki67 and p65 expressions	50 µM (in vitro) 8 mg/kg/2 days for two weeks (in vivo)	[84]
Baicalein *Scutellaria baicalensis*	Flavone	In vitro and in vivo EBV-positive NPC cells	Tumor development. Sp1, EBNA1, and EBNA1-Q-promoter expressions	15, 30, and 60 µM (in vitro) 30 mg/kg/day for 21 days (in vivo)	[85]
		In vitro EBV-positive B-cell	Tumor growth CD74 and CD44 expressions	100 µM	[86]
Quercetin Diverse fruits, vegetables, and medicinal herbs	Flavonol	In vivo EBV-gastric carcinoma cells	Tumor growth. EBNA1 and LMP2 expressions	30 mg/kg/day for 2 weeks	[87]
		In vivo EBV-gastric carcinoma cells	Tumor growth. EBNA1 and LMP2 expressions	10 mg/kg/23 days (*Ganoderma lucidum* extract) 10 mg/kg/23 days (quercetin)	[88]
		In vitro EBV-gastric carcinoma cells	Tumor growth. Entry and latent phases of EBV EBNA1 expression	62 µM	[77]
		In vitro EBV-positive Burkitt’s lymphoma cells	Tumor growth c-Myc expression	100 µM	[89]
		In vitro EBV-positive NPC cells	Cell proliferation FASN expression	100 µM	[90]
		In vitro EBV-associated lymphoma cells	Tumor development IL-6 expression	10 µM	[91]
Icaritin *Epimedium* genus	Flavonol	In vitro EBV-associated ENKTCL	Tumor development Cell proliferation Bcl-2, pBad, and LMP1 expression	16–50 µM	[92]
Fisetin Diverse fruits, vegetables, and medicinal plants	Flavonol	In vitro EBV-infected NPC cells	Tumor growth and metastasis LMP1, vimentin, and twist proteins expression	12.5–100 µM	[93]
		In vitro EBV-infected NPC cells	Tumor growth. LMP1, p65, IκBα, and CyclinD1 proteins expression	6.25–100 µM	[94]
Dihydromyricetin (ampelopsin) *Ampelopsis grossedentata*	Dihydroflavonol	In vitro EBV-positive Burkitt’s lymphoma cells	Tumor development. Cell proliferation. LMP1-associated pathway	0.1–50 µM	[95]

Abbreviations: B-cell, B lymphocyte; Bcl-2, anti-apoptotic protein; c-Myc, cellular myelocytomatosis oncogene; CyclinD1, a protein involved in the regulation of the cell cycle; EBNA1, Epstein–Barr nuclear antigen 1; EBV, Epstein–Barr virus; ENKTCL, extranodal natural killer (NK)/T-cell lymphoma (ENKTCL); FASN, fatty acid synthase; IL-6, interleukin-6; IκBα; an inhibitor of κB alpha, effectively inhibiting the activity of the nuclear factor-κB; LMP1, latent membrane protein 1; LMP2, latent membrane protein 2; NPC, nasopharyngeal carcinoma; p65, a protein that serves as a subunit of the nuclear factor-κB transcription factor; pBad, protein Bad; Rta, replication and transcription activator; SP1, transcription factor specificity protein 1; SREBP1, sterol regulatory element-binding protein 1; Zta, an immediate-early gene.

## Data Availability

The manuscript comprehensively includes all pertinent data.
